# A diet-change modulates the previously established bacterial gut community in juvenile brown trout (*Salmo trutta*)

**DOI:** 10.1038/s41598-019-38800-7

**Published:** 2019-02-20

**Authors:** Stéphanie C. Michl, Matt Beyer, Jenni-Marie Ratten, Mario Hasler, Julie LaRoche, Carsten Schulz

**Affiliations:** 1 Gesellschaft für Marine Aquakultur mbH (GMA) Büsum, Büsum, Germany; 2 Department of Marine Aquaculture, Institute of Animal Breeding and Husbandry, Christian-Albrechts-Universität zu Kiel, Kiel, Germany; 3 GEOMAR Helmholtz Centre for Ocean Research Kiel, Kiel, Germany; 4 Department of Biology, Dalhousie University, Halifax, Canada; 5 Lehrfach Variationsstatistik, Christian-Albrechts-Universität zu Kiel, Kiel, Germany

## Abstract

The aim of the present study was to investigate the impact of dietary plant proteins on the gut microbiome of first feeding brown trout (*Salmo trutta*) reproduced from wild stocks and to evaluate whether the initial microbiome of brown trout fry can be permanently manipulated by the first feeding diet. Therefore, brown trout fry was fed diets based on either 0%, 50% or 90% plant-derived proteins from first feeding onwards and via 16S rRNA gene sequencing a strong dietary influence on the bacterial gut community on phylum and order level was detected. Proteobacteria and Fusobacteria were significantly enhanced when fishmeal was integrated into the experimental diet, whereas plant-derived proteins significantly promoted Firmicutes and Bacteroidetes. In order to evaluate whether the first feeding diet had a permanent effect on the initially established microbial gut community of juvenile brown trout, a cross-over diet-change was applied 61 days post first feeding. 48 days after the diet-change, the gut microbiome of all dietary groups was significantly different from the one initially established after first feeding. Moreover, the first feeding diet had no statistically significant influence on the gut microbiome after the diet-change, demonstrating no permanent effect on the gut microbiome formation.

## Introduction

In freshwater aquaculture, rainbow trout (*Oncorhynchus mykiss*) is preferentially used for the production of consumable fish compared to brown trout (*Salmo trutta*). Brown trout has a significantly longer hatching period and exhibits slower growth than rainbow trout when exposed to the same rearing conditions^1^. Nevertheless, the production of brown trout is of high commercial interest for recreational angling and restocking purposes. In modern salmonid feeds, fishmeal has been significantly substituted by plant-derived proteins due to the declining availability of fishmeal and increasing prices^2^. The usability of plant-based diets for salmonids has been evaluated in several studies and for different life stages^3,4^, but mainly for Atlantic salmon (*Salmo salar*) or rainbow trout. The inclusion of plant-derived proteins can highly impact physiology, growth performance and health of fish^5–7^. An essential element of digestion and health is the microbiome^8^ and despite an increasing knowledge about the relationship between the intestinal bacterial community and its host^9–11^, very little is known about specific dietary effects on the microbiome of juvenile brown trout. The impact of plant-based diets on the microbial community of rainbow trout^12^ and Atlantic salmon^13,14^ has been investigated previously. In rainbow trout fry it was also demonstrated that first feeding initiates the gut microbiome establishment and that diet-type influences the bacterial composition^15^. Our own research about the impact of plant-based proteins on the microbiome of rainbow trout fry furthermore revealed that the established gut microbiome at first feeding is highly malleable and that the bacterial community structure reflects the actual diet fed at the time of sampling^16^. Nevertheless, the intestinal microbiota of vertebrates is not only influenced by environment, diet, health status or stress^8^ but also by host genetics^17^. For example, certain bacterial groups of the rainbow trout gut microbiota significantly correlate with individual trout families and the dietary effect on the bacterial community structure can be influenced by the individual family as well^18^. Hence, the genetic background of juvenile brown trout (often originating from wild stocks) could affect the influence of dietary plant proteins on the bacterial gut community differently, compared to rainbow trout of well-established breeding lines. We therefore aimed to investigate the impact of plant-derived dietary proteins on the intestinal microbiome of juvenile brown trout (*Salmo trutta*) reproduced from wild brown trout. We additionally evaluated whether the early-established gut community of trout fry is permanently shaped by the first feeding diet. In order to evaluate this hypothesis, three experimental diets with varying inclusion-levels of plant-derived proteins were fed from first feeding on and subsequently changed in a cross-over feeding design. The bacterial gut community was investigated via sequencing of the 16S rRNA gene regions V6-V8.

## Material and Methods

### Experimental animals

The present experiment was conducted at the “Gesellschaft für Marine Aquakultur mbH” (Büsum, Germany). Eyed brown trout eggs (*Salmo trutta*) were reproduced and bred from wild brown trout caught in Schleswig-Holstein (Germany) at the “Fischbrutanstalt Altmühlendorf” (Germany). All animal handling procedures were approved by the animal welfare officer of the “Gesellschaft für Marine Aquakultur mbH” and the local authority of Schleswig-Holstein according to the German animal welfare law (TierSchG).

### Experimental setup

**Figure 1:**
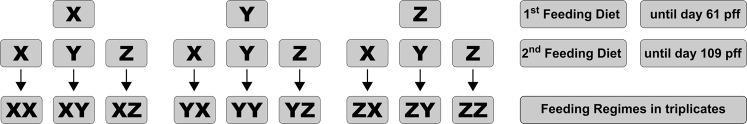
Experimental design. The scheme visualises the experimental design used in the present feeding trial. From first feeding until 61 days post first feeding (dpff) fish were fed one of the three 1^st^ Feeding Diets without replication. After dpff 61 all experimental diets were changed in a cross-over design and until 109 dpff fish were fed in triplicate one of the 2^nd^ Feeding Diets. All possible combinations of 1^st^ and 2^nd^ Feeding Diets resulted in the nine final Feeding Regimes. Modified after Michl *et al*.^20^.

Three isonitrogenous and isoenergetic diets (diet X, diet Y and diet Z; Table 1) with different plant protein inclusion levels (0%, 50% and 90%, respectively) were formulated and produced in cooperation with Skretting ARC (Aquaculture Research Center; Stavanger, Norway). Each diet was formulated in accordance with the NRC^19^ digestible amino acid requirements for small Atlantic salmon fry (0.2–20.0 g) and also the composition of the vitamin and mineral premixtures were according to the NRC (2011) guidelines. 6000 eyed trout eggs were randomly distributed among three commercial hatching troughs (2000 eggs each) integrated into a recirculating freshwater waterbody. Until hatching day, average water temperature was 11.2 ± 0.3 °C. Throughout the experiment, average pH was 8.1. Fish were reared in the recirculating system for a total of 143 days and fed the three experimental diets from first feeding on. Feed was provided for the first time 20 days post hatch (dph), but active first feeding of trout fry was observed 28 days post hatch. Each of the three experimental diets was provided to the fish of one hatching trough, without replication, resulting in the 1^st^ Feeding Diet groups X, Y and Z (Fig. 1). Feed was supplied *ad libitum* by automatic feeders once per hour for about six weeks, which was then gradually reduced until four times per day. Dimmed light was provided from 06.00 to 21.00 hours. 61 days post first feeding (dpff) diets were changed in a cross-over feeding design to investigate possible nutritional programming effects of the 1^st^ Feeding Diet. 1440 trout fry from each of the three hatching troughs were randomly distributed among nine 50 L aquaria integrated in the established recirculating system, resulting in a total of 27 aquaria. Average temperature of the waterbody was 12.4 ± 1.3 °C until the end of the experiment. All diets were changed in a cross-over design (see Fig. 1) and until day 109 pff each experimental group was fed four times per day their 2^nd^ Feeding Diet with 2.2% of the total biomass per day. All second feeding diets were applied in triplicates. Although it was almost impossible to measure feed intake directly in such small fish, care was taken at feeding to ensure that all fish have eaten the applied feed portion.

**Table 1:** Composition of experimental diets.

Experimental diets	Diet X	Diet Y	Diet Z
Ingredients (in % of dry matter)			
Fishmeal	77.6	33.5	11.0
Corn gluten		10.0	16.7
Sunflower meal		4.5	3.1
Soy protein concentrate		15.0	20.0
Wheat gluten		14.2	25.0
Faba bean meal		4.5	2.0
Wheat starch	13.0	5.0	5.0
Vitamin & Mineral Premixtures	0.7	1.0	3.1
Fish oil	8.7	12.3	14.1
Proximate composition (in % of dry matter)			
Dry matter (in % of diet)	93.4	94.7	92.9
Crude protein	57.3	58.3	57.6
Crude fat	18.0	18.7	18.8
Crude ash	9.5	6.2	4.6
Gross energy (MJ kg^−1^)	23.1	24.0	24.3
Amino acid composition (in % of diet)			
Arginine	2.4	3.2	2.9
Histidine	1.0	1.2	1.2
Isoleucine	2.0	2.1	2.1
Leucine	4.4	3.9	4.3
Lysine	2.5	3.6	2.7
Methionine	0.9	1.4	1.1
Cystine	0.8	0.5	0.7
Phenylalanine	2.5	2.0	2.4
Tyrosine	1.0	1.3	1.4
Threonine	1.6	2.2	1.9
Valine	2.1	2.5	2.4
Alanine	2.4	3.0	2.7
Aspartic acid	3.5	4.5	4.2
Glutamic acid	12.2	7.9	10.6
Glycine	1.9	3.0	2.4
Proline	4.0	2.5	3.4
Serine	2.4	2.3	2.5
Fatty acid composition (in % of total fatty acids)			
n-6/n-3 ratio	0.5	0.2	0.4
Total n-6	10.1	4.0	7.6
Total n-3	19.5	23.7	21.6
ALA/LA ratio	0.2	0.4	0.2
Total C18:2n-6 (LA)	9.1	2.8	6.4
Total C18:3n-3 (ALA)	1.6	1.0	1.2
EPA/DHA ratio	0.9	0.7	0.8
Total C20:5n-3 (EPA)	6.5	7.6	7.2
Total C22:6n-3 (DHA)	7.3	11.1	9.1

### Sampling

For microbiome analysis, 150 fish were sampled in total. Fish were starved for 12 hours before sampling. Experimental animals were anaesthetized with MS222 (Tricaine methanesulfonate, E10521, Sigma-Aldrich Co. LLC.) and immediately killed by cutting the gill vein. Five animals from each of the three hatching troughs were sampled on day 61 pff (15 animals in total) and five animals from each aquarium (135 animals in total; 3 aquaria per treatment = 15 animals per treatment) were sampled on day 109 pff. The whole digestive tract was dissected on ice using sterile razor blades and instantly frozen at −80 °C. The growth performance of fish was monitored via dry and wet body weights during the course of the whole experiment and data were recently published and discussed in Michl *et al*.^20^.

### DNA extraction

The Qiagen DNeasy^®^ Blood & Tissue DNA extraction kit was used according to the manufacturer’s specifications to extract DNA from tissue samples. Digestive tract samples were thawed at 4 °C and homogenised (KT Miccra D9 homogenizer) on ice in 1 ml of a 5 mg ml^−1^ lysozyme (8259, Carl Roth) in TE-buffer solution (10 mM Tris-HCl, 1 mM EDTA) for 30 seconds. The homogenised solution was incubated for 30 min at 37 °C. Afterwards, the homogenate was gently vortexed and 80 µl were incubated for 60 min at 56 °C in 200 µl of lysis buffer AL (provided in the extraction kit), 20 µl Proteinase K and 100 µl PBS (Solution without Ca-Mg, 733–2296, VWR). After incubation, 200 µl ethanol (96–100%) was added and further extraction steps were performed according to the manufacturer’s protocol for purification of total DNA from animal tissue. Two extra washing steps with the provided buffers AW1 and AW2 were included into the protocol, as well as an extra centrifugation step of 1 min at maximum speed before elution, according to recommendations by Qiagen. For purification, extracted DNA was incubated with RNase A (Qiagen) (1 mg ml^−1^ in DEPC water), pretreated by an inactivation of remaining microbial DNases at 70 °C for 15 min. The RNase A working solution was added to each sample to a final concentration of 100 µg ml^−1^ RNase A and incubated for 30 min at 60 °C. A final DNA clean-up step was performed using the NucleoSpin^®^ gDNA clean-up kit (Machery-Nagel) by following the manufacturer’s protocol including all recommended steps. DNA concentrations after the clean-up step were measured via the NanoDrop^TM^ UV-Vis Spectrophotometer (Thermo Fisher Scientific). Average concentrations were 41.8 ngµl^−1^ ± 47.0 (min 3.3 ngµl^−1^, max 229.0 ngµl^−1^, median 25.1 ngµl^−1^) with average 260/280 ratios of 2.05 ± 0.27 (min 1.80, max 3.32, median 1.96).

### 16S rRNA gene PCR amplification and sequencing

DNA amplification and sequencing were performed by PCR targeting the V6-V8 region of the 16S rRNA gene in a two-step-procedure, as it was difficult to amplify the 16S rRNA gene from brown trout gut samples. Step one: the final PCR reaction volume was 20 µl including 4 µl 5X Phusion GC buffer, 0.6 µl concentrated DMSO, 0.4 µl dNTP (10 mM), 0.4 µl of each primer (25 µM), 0.2 µl Phusion high-fidelity polymerase (2 Uµl^−1^; Thermo Fisher Scientific Inc.), 12 µl DEPC H_2_O and 2 µl of DNA template following the Phusion high-fidelity polymerase standard protocol. Primers used were B969F (5′-ACG CGH NRA ACC TTA CC-3′) and BA1406R (5′-ACG GGC RGT GWG TRC AA-3′) from IDT (Integrated DNA Technologies, Inc.) according to Comeau *et al*.^21^. Cycling protocol was as follows: 98 °C for 3 min, 35 cycles of 98 °C for 10 sec., 54 °C for 30 sec. and a final extension at 72 °C for 1 min, and finally 72 °C for 10 min. Results of the PCR were verified on a 1.1% agarose gel. DNA samples were stained with SYBR safe DNA gel stain (Invitrogen^TM^, Thermo Fisher Scientific Inc.) and images were analysed using a gel imaging box (G:BOX, Syngene). Due to purchasing issues, one third of the samples were amplified with the Phusion Hot Start II polymerase (Thermo Fisher Scientific Inc.). The change of polymerases had no statistically significant effect on the results as verified via PCA-ANOVA approach (see detailed description below). The cycling protocol for samples amplified with the Phusion Hot Start II polymerase was as follows: 98 °C for 30 sec, 35 cycles of 98 °C for 10 sec, 54 °C for 30 sec and a final extension at 72 °C for 30 sec, and finally 72 °C for 10 min. The purification of PCR samples from the gel was performed by carefully inserting a micropipette tip into the band and slowly drawing the DNA-loaded agarose plug into the end of the tip. The agarose plug was then released into a well of a 96-well plate to diffuse out into 20 μl of DEPC water overnight in the refrigerator (approx. 4 °C). Step two: an Illumina MiSeq platform was used to sequence the amplified 16S rRNA gene fragment at the Integrated Microbiome Resource lab (IMR) at Dalhousie University (Halifax, Canada) following the procedure described in detail by Comeau *et al*.^22^. The samples were multiplexed at equal volumes with custom 16S fusion primers. The fusion primers contained Illumina Nextera adapters and barcodes for dual-indexing at both the forward and reverse paired ends of the fragments. Amplifications were performed using two different dilutions (undiluted and 1:10). 25 µL reactions contained: 5 µL of 5xHF PCR Buffer, 0.5 µL dNTPs (40 mM), 5 µL forward and 5 µL reverse primer (1 µM), 0.25 µL Phusion polymerase (2 U µL^−1^; Thermo Scientific), 2 µL template and 7.25 µL PCR-grade water. Cycling conditions were: 98 °C (30 s), followed by 30 cycles of 98 °C (10 s), 55 °C (30 s) and 72 °C (30 s). Final extension was performed for 4.5 min at 72 °C. The PCR product quality was verified using the E-gel 96-well high-throughput system (Invitrogen^TM^, Thermo Fisher Scientific Inc.). Amplicons were cleaned up and normalized simultaneously via the high-throughput Invitrogen SequalPrep 96-well plate kit (Invitrogen^TM^, Thermo Fisher Scientific Inc.). The samples and negative controls were pooled into one library. The library was quantified with Qubit (Invitrogen^TM^, Thermo Fisher Scientific Inc.) and loaded into the Illumina MiSeq platform as a 20 pM final denatured library according to the manufacturer’s protocol using 2 × 300 bp PE v3 chemistry suitable for overlap and stitching together of paired amplicon reads into one full-length read of higher quality^22^.

### Bioinformatics

The analysis of raw sequences was performed with QIIME (Quantitative Insight Into Microbial Ecology) for the analysis of high-throughput community sequencing data^23^. The analysis was performed by following the steps of the 16S amplicon analysis procedure of the Integrated Microbiome Resource lab (IMR)^24^. First, several quality control steps were applied: forward and reverse reads were stiched using PEAR (Paired-end rEAd merger^25^); low quality reads (<Q30 over 90% of length and minimum size of 400 bp) were removed with FASTX-Toolkit and BBMap; sequences with unidentified nucleotides, with mitochondrial and chloroplast DNA sequences^24^, and chimeric DNA molecules were removed (using UCHIME^26^). Second, open-reference OTU (Operational Taxonomic Units) picking was performed against the Greengenes reference database^27^ using *sortmerna* and *sumaclust* for the de novo portion^28^. OTUs were grouped together based on 97% sequence identity. Low-confidence (i.e. MiSeq bleed-through) OTUs were subsequently removed, with the threshold for removing low confidence reads being set to 0.1%. This had been reported by Illumina to be the maximum of bleed-through reads on the Illumina MiSeq platform. The collection of sequences was rarefied to 1000 reads per sample, which had been suggested for gut samples by Hamady & Knight^29^ and which allowed a sufficient number of samples from each tank to remain for statistical analysis. In total, 122 samples remained after the quality steps during the bioinformatics workflow and were incorporated into the final statistical analysis. Please see Table 2 for a summary of those samples.

**Table 2:** Overview of the sample numbers used for final statistical analysis.

Dpff	61			109																										
Treatment	X	Y	Z	XX			XY			XZ			YX			YY			YZ			ZX			ZY			ZZ		
Replicate	I	I	I	I	II	III	I	II	III	I	II	III	I	II	III	I	II	III	I	II	III	I	II	III	I	II	III	I	II	III
No. of fish	5	3	4	3	4	3	4	5	3	5	4	5	5	4	1	4	4	3	4	4	4	5	5	5	5	3	4	4	5	5

Presented is the number of samples per treatment and replicate tank that remained after the quality steps during the QIIME workflow and were integrated into the final statistical analysis (one hatching trough per treatment on sampling day 61 pff and three replicate tanks per treatment on sampling day 109 pff). The experimental diets are X: 0% plant proteins, Y: 50% plant proteins, Z: 90% plant proteins.

### Statistics and Sample size

The required number of fish necessary for this study was estimated based on a Monte-Carlo simulation in the statistical software R (version 3.4.1^30^), and pre-approved by the animal welfare officer of the “Gesellschaft für Marine Aquakultur mbH” and the local authority of Schleswig-Holstein according to the German animal welfare law (TierSchG). Further, we estimated the statistical power and necessary sample size for finding significant differences in relative abundances of bacterial taxa between treatments based on an ANOVA, rather than assumed *a priori* variances of the data in the context of Nonmetric multidimensional scaling (NMDS) or Principal Component Analysis (PCA). From published data on the microbiome of juvenile rainbow trout^15^ we hypothesized a difference in means of 0.67 with homoscedastic data. The simulation achieved a statistical power of 0.91 with a given sample size of n = 12 per treatment and a two-sided significance level of 0.05. These analyses led us to use 15 animals per treatment, in order to cover a potential loss of up to 20% of samples for microbiome analysis.

The number of observed distinct OTUs was evaluated via rarefaction curves, the Chao1 richness estimator and the Shannon diversity index H’ were calculated based on the OTU table generated during the QIIME workflow for estimating alpha diversity. Nonmetric multidimensional scaling (NMDS) was performed to graphically explore differences between bacterial communities on order level in relation to the dietary treatment or sampling day using the R package vegan^31^. Data was Hellinger-transformed and analysed by a Bray-Curtis dissimilarity matrix. The stress factor was calculated to estimate the representation of original data in the ordination space.

First, the impact of the experimental diets X, Y and Z on alpha diversity and the top-five bacterial phyla with the highest relative abundance during the first feeding period was tested with a statistical model based on generalized least squares^32^, with first-feeding diet considered as fixed factor. Based on a graphical residual analysis, data were assumed to be approximately normally distributed and to be heteroscedastic. An analysis of variances (ANOVA) was conducted and in order to compare the first-feeding diets, multiple contrast tests were performed^33^ using the R package SimComp^34^.

Second, the impact of the nine feeding regimes on alpha diversity and the top-five phyla with the highest relative abundance at the end of the second feeding period was tested. A statistical mixed model was established^35,36^ with the 1^st^ Feeding Diet and the 2^nd^ Feeding Diet as well as their interaction term as fixed factors. The data were assumed to be approximately normally distributed and heteroscedastic. Aquaria were defined as random factor. Based on this model, an ANOVA was conducted, followed by multiple contrast tests in order to compare the levels of the fixed factors^37,38^ using the R package multcomp^39^. A nutritional programming effect of the intestinal microbiome was considered when all of the following three assumptions were met simultaneously: i) a significant effect of the 1^st^ Feeding Diet, ii) a non-significant effect of the 2^nd^ Feeding Diet and iii) a non-significant interaction of the 1^st^ and the 2^nd^ Feeding Diet. Data were pooled for the 1^st^ Feeding Diet, in case of a non-significant interaction only, and multiple contrast tests were rerun to compare only the three 2^nd^ Feeding Diets (X, Y and Z).

Third, statistical differences of alpha diversity indices and bacterial phyla between the two sampling points (61 and 109 dpff) were tested for the feeding regimes XX, YY and ZZ. A statistical mixed model was established with sampling day as fixed factor and tank as random factor. An ANOVA was conducted, followed by multiple contrast tests to compare the two sampling days as described previously^37,39^.

Fourth, the impact of the experimental diets on the bacterial community structure during the first-feeding period was tested. Therefore, a Principal Component Analysis (PCA) was performed^40^ with Hellinger-transformed bacterial order relative abundances. Order level was used as a compromise between a precise (necessary sequencing depth) and a robust statistical analysis. Those principal components (PC) from the PCA with the greatest influence on data variability were selected for further analysis by using the Broken-Stick-Criterion^41^. Based on the first two PCs, rotated data (i.e. pseudo-variables) were calculated and integrated into a multivariate model, established simultaneously for the two pseudo-variables. Based on this model, an ANOVA was performed. Multiple contrast tests for multiple endpoints were conducted in order to compare the experimental diets simultaneously for the two pseudo-variables^34,42^.

Fifth, the impact of the nine feeding regimes on the bacterial community structure at the end of the second feeding period was evaluated. A PCA was performed with Hellinger-transformed relative abundance data on order level and the PCs with the highest influence on data variability were selected as described previously. The first six PCs represented 82% of the cumulative variance. Based on these six PCs, rotated data were calculated and integrated into a multivariate mixed model, established simultaneously for the six pseudo-variables. The 1^st^ Feeding Diet and the 2^nd^ Feeding Diet as well as their interaction term were considered as fixed factors, the tanks as random factor. Based on this model, an ANOVA was conducted. A nutritional programming effect was defined by the previously established assumptions. Finally, multiple contrast tests for multiple endpoints were performed to compare the levels of the fixed factors simultaneously for the six pseudo-variables^34,42^.

Sixth, the first two PCs were further examined for the individual contribution of specific bacterial orders to the cumulative variance explained of each principal component. In case of a non-significant interaction of the first and the second feeding diet in the previous model, data were pooled for the first-feeding diet and multiple contrast tests as described before^34,42^ were performed to compare the three second feeding diets (X, Y and Z) simultaneously for the top-ten orders with the highest loadings on each of the two PCs, respectively for each PC. Thus, specific bacterial orders significantly promoted by a certain diet-type were identified.

Seventh, statistical differences of the bacterial community structure between the two sampling days (61 and 109 dpff) were evaluated for continuously fed fish (feeding regimes X and XX, Y and YY, Z and ZZ, respectively). PCA was performed for each of the three Hellinger-transformed data pairs and the first three PCs were selected as described before. Resulting pseudo-variables were integrated into a multivariate mixed model established simultaneously for the three pseudo-variables. The sampling day was integrated as fixed factor and tanks as random factor. An ANOVA was conducted, followed by multiple contrast tests for multiple endpoints to compare the two sampling points simultaneously for the three pseudo-variables as described before^34,42^.

## Results

### Growth performance

The growth performance of individual fish was extensively monitored during the course of the experiment and results were presented in a companion study on digestive enzyme activity from the same experimental set up (Michl *et al*.^20^). Wet body weights measured 61 days post first feeding were statistically equal between the treatment groups X, Y and Z. 109 days post first feeding, fish fed the 2^nd^ Feeding Diet X after the diet change had significantly higher wet body weights when compared to the 2^nd^ Feeding Diets Y and Z. The 1^st^ Feeding Diet Y promoted significantly higher wet body weights when compared to those achieved on diet X when fish were fed the 2^nd^ Feeding Diets X and Y. Trout continuously fed diet Z (treatment ZZ) had significantly reduced wet body weights compared to treatments XX and YY.

### Alpha diversity

**Figure 2:**
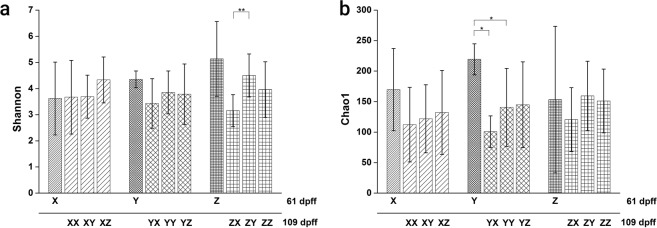
Alpha diversity indices in relation to dietary treatment and sampling point. Presented are (**a**) the Shannon diversity index and (**b**) the Chao1 richness estimator as means (SD) of individual fish per treatment and sampling day (data was obtained from one tank per treatment on day 61 pff and three tanks per treatment on day 109 pff; please see Table 2 for exact sample size). Significant differences between treatments are indicated by asterisks: P < 0.05 (*), P < 0.01 (**).

As it was difficult to amplify the 16S rRNA gene from Brown trout samples, sequences were rarefied to 1000 reads to make a compromise between the quality scores for the sequences and the fact that an appropriate number of samples remains for statistical analysis (see rarefaction curves; Supplementary Figure S1).For all individual fish Shannon diversity indices and Chao 1 richness estimators have been calculated. In Fig. 2a the only statistically significant difference between Shannon diversity indices can be observed between treatments ZX and ZY. The Chao1 richness estimator was also not generally affected by the diet type, except for the 1^st^ Feeding Diet Y. As can be seen in Fig. 2b, richness decreased significantly from day 61 pff to day 109 pff, when diets X or Y were fed during the second feeding period. The multivariate ANOVA revealed no significant interaction between the first and the second feeding diet for the two indices and hence data were pooled for the 1^st^ Feeding Diet. Overall, significantly decreased Shannon diversity indices were observed when diet X was fed as 2^nd^ Feeding Diet (*P* < 0.05).

### Relative abundance of bacterial phyla

**Figure 3:**
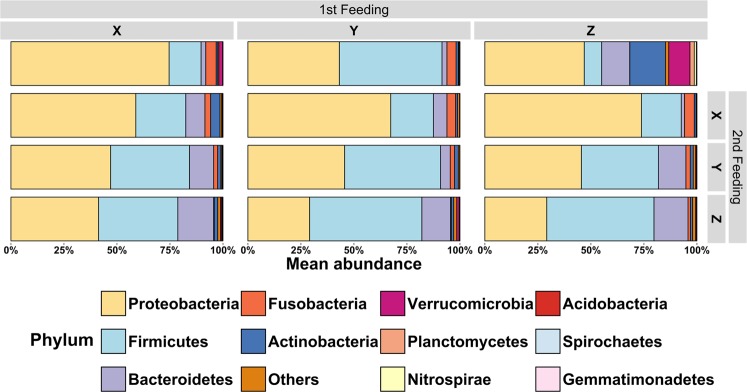
Mean relative abundance of phyla in relation to the dietary treatment and feeding period. The graph shows the mean relative abundance in percent of phyla that are present in ≥10% of all samples and account for ≥1% of all phyla. Phyla that did not fulfil those conditions were combined into “Others”. The data presented are means calculated from individual fish (one tank per treatment at the end of the first-feeding period (61 dpff); three tanks per treatment at the end of the second feeding period (109 dpff); please see Table 2 for exact sample size).

The most relative abundant phyla present in the GI tracts of fish were analysed with regard to dietary influences (see Supplementary Table S1). Figure 3 visualises the relative abundance of phyla that are present in at least 10% of all samples and accounting for at least 1% of all observed OTUs. For all treatments Proteobacteria and Firmicutes were the dominant phyla, followed by Bacteroidetes and Fusobacteria. The diet type had a significant influence on the relative abundance of most phyla, in contrast to the sampling point which is shown for the top-five most abundant phyla in Table 3. Fusobacteria significantly decreased from the first to the second feeding period when diet Y was fed continuously, but significantly increased when diet Z was fed in both feeding periods. The largest difference between sampling points can be observed for Firmicutes in fish fed diet Z: the relative abundance increased from 8% at the end of the first feeding period to 51% at the end of the second feeding period. The diet change applied at the end of the first feeding period had a significant effect on the relative abundance of Proteobacteria and Firmicutes in fish of the 1^st^ Feeding Diet groups Y and Z. In fish of treatment X however, none of the phyla were affected by the diet change and remained relatively constant until the end of the second feeding period. The statistical analysis indicated no significant interaction between the first and the second feeding period, however, the results demonstrated that Proteobacteria were significantly enhanced when fishmeal was integrated into the second feeding diet (*P* < 0.01). The same findings can be observed for Fusobacteria (*P* < 0.01). Firmicutes, in contrast, were significantly promoted by plant proteins (*P* < 0.001), and so were Bacteroidetes (*P* < 0.05). The phylum Actinobacteria, however, was not affected by the diet type at all. In Fig. 3 it can be additionally observed that in fish of treatment Z the number of phyla and their relative abundance are higher compared to the treatments X and Y, which aligns well with the results of the diversity analysis presented in Fig. 2.

**Table 3:** The top five most abundant phyla in relation to the dietary treatment.

Diet	dpff	Proteobacteria		Firmicutes		Bacteroidetes		Fusobacteria		Actinobacteria	
		Mean	SD	Mean	SD	Mean	SD	Mean	SD	Mean	SD
**Mean abundance of phyla in [%]**											
X	61	74.5^a^	9.9	15.1^a^	11.8	2.2	0.4	4.9^a^	3.0	0.8	0.3
XX	109	58.9	24.3	23.6	24.2	9.0	16.2	2.7	2.5	4.3	10.1
XY	109	47.0	11.7	37.2	16.3	11.4	22.7	1.9	1.6	1.5	1.2
XZ	109	41.3	23.7	37.4	31.1	16.9	18.5	0.3	0.5	1.6	1.2
Y	61	43.1^b^	2.4	48.4^b^	1.0	2.3	1.9	4.3^a,b,*^	1.1	1.1	0.3
YX	109	67.4^A^	22.6	20.1^A^	14.2	6.4	9.1	4.1^A^	2.8	0.9	0.8
YY	109	45.6^A,B^	17.1	45.2^B^	20.2	4.5	9.0	2.0^A,*^	1.7	1.8	2.8
YZ	109	29.1^B^	20.4	53.0^B^	31.0	13.5	19.6	0.4^B^	0.4	1.1	1.0
Z	61	46.9^a,b^	18.7	8.2^a,*^	7.2	13.2	8.8	0.1^b,*^	0.2	16.8	23.6
ZX	109	73.9^A^	9.0	18.8^A^	7.4	1.6	1.5	4.6^A^	2.7	0.9	1.8
ZY	109	45.5^B^	10.6	36.3^A,B^	20.3	13.0	16.1	2.0^B^	1.2	1.4	1.0
ZZ	109	29.2^C^	10.7	50.6^B,*^	26.2	16.0	18.8	1.2^B,*^	1.5	1.0	1.0

The average relative abundance in percent of the top five most abundant phyla are presented (fish were sampled from one tank per treatment at the end of the first-feeding period (61 dpff); and from three tanks per treatment at the end of the second feeding period (109 dpff); please see Table 2 for exact sample size and Supplementary Table S1 for detailed information).

^a,b,c^Statistically significant differences (*P* < 0.05) between first-feeding diets are indicated by lower case superscript letters.

^A,B,C^Statistically significant differences (*P* < 0.05) between second feeding diets are indicated by upper case superscript letters, separate for each corresponding first-feeding diet.

*Statistically significant differences (*P* < 0.05) between sampling points of continuously fed diets are indicated by superscript asterisks.

### Shaping the gut microbiome

**Figure 4:**
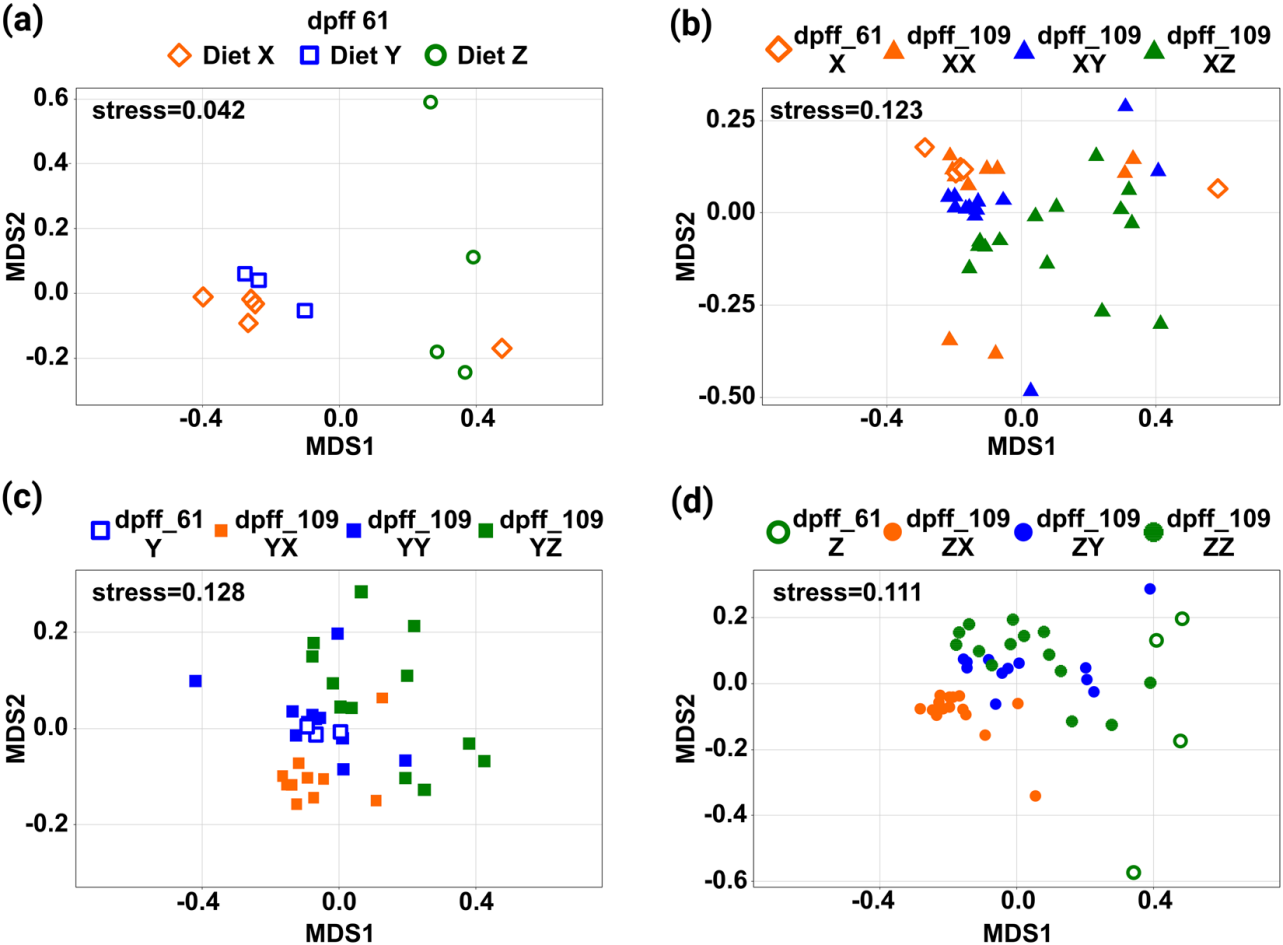
Nonmetric multidimensional scaling (NMDS) of the bacterial communities. Presented are ordination plots based on Bray-Curtis-distances between samples (calculated with relative abundances of bacterial orders). Each point in the two-dimensional space represents an individual fish and the distance between points represents the dissimilarity of the respective microbiomes. Panel (a) shows the bacterial community structure of dietary treatments X, Y and Z on day 61 pff. Panels (b–d) present the bacterial community structure of the 1^st^ Feeding Diets on day 61 pff in relation to the corresponding 2^nd^ Feeding Diets on day 109 pff after the diet change. The stress level is a qualitative indicator of the original data representation. The shape of points refers to the 1^st^ Feeding Diet; the colours indicate the 2^nd^ Feeding Diet. Open objects are samples obtained on day 61 pff.

**Figure 5:**
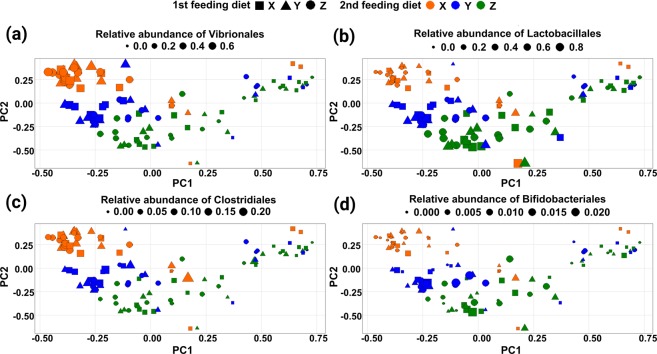
PCA plots of the intestinal microbiome. The four panels show the bacterial community at the end of the second feeding period, represented by pseudo-variables generated during Principal Component Analysis in relation to the 1^st^ and 2^nd^ Feeding Diets. Each object represents one individual fish. Additionally, the panels (a–d) present the relative abundances of Vibrionales, Lactobacillales, Clostridiales and Bifidobacteriales, respectively, for each fish. The shape of objects represents the 1^st^ Feeding Diet; the colours indicate the 2^nd^ Feeding Diet and the size of each object relates to the relative abundance of the bacterial order that is indicated in the legend of each panel.

Nonmetric Multidimensional Scaling (NMDS) was performed to graphically explore the bacterial communities on order level between the different treatments and over the course of the experiment (Fig. 4). The stress levels of all four NMDS plots are below 0.13, indicating a good representation of the original data. Figure 4a reveals that the microbiomes after the first feeding period of fish fed the fishmeal diet X and the mixed diet Y are similar, but the microbiome of fish fed the plant-based diet Z is different. Furthermore, it can be seen in Fig. 5b–d that the microbiomes of fish group by the second feeding diet after the diet change. In general, data points representing fish fed the plant-based second feeding diet Z are more diffuse, compared to diets X and Y. The microbiomes of fish continuously fed either diet X or Y (Fig. 4a,b) are very similar between day 61 pff and day 109 pff. However, the microbiomes of fish continuously fed diet Z alter from the first to the second feeding period (Fig. 4d).

The observed alterations in the microbial gut community were further statistically explored via Principal Component Analysis (PCA) and a subsequent multivariate ANOVA: At the end of the first feeding period the bacterial order composition was only significantly different between fish fed diets Y and Z (*P* = 0.003), which was probably affected by the small sample size as well. The ten bacterial orders with the highest influence on separating the two treatments were Vibrionales, Rhodobacterales, Lactobacillales, Clostridiales, Rhodospirillales, Rhizobiales, Verrumicrobiales, Fusobacteriales, Alteromonadales and Saprospirales (see Supplemental Table S1). At the end of the second feeding period neither a significant influence of the 1^st^ Feeding Diet on the bacterial order composition, nor a significant interaction between the 1^st^ and the 2^nd^ Feeding Diet could be detected, indicating no permanent effect of the 1^st^ Feeding Diet on the intestinal microbiome formation of trout fry. Nevertheless, the influence of the 2^nd^ Feeding Diet on the bacterial order composition was highly significant (*P* = 0.001), and thus data were pooled for the 1^st^ Feeding Diet for subsequent analysis. According to the PCA-ANOVA analysis the 2^nd^ Feeding Diet X was significantly separated from the other two diets by PC2 (*P* = 0.000; Table S1) and the 2^nd^ Feeding Diet Z was separated from X and Y by PC1 (*P* = 0.000). The analysis of the bacterial orders contributing to the principal components indicated a strong influence of Vibrionales, Alteromonadales, Lactobacillales and Bifidobacteriales in separating fish fed the fishmeal diet X from the other diets. The plant-based diet Z in contrast is separated from the other diets by the relative abundance of Vibrionales, Lactobacillales, Alteromonadales, Clostridiales, Fusobacteriales and Saprospirales (amongst others). The relative abundance of those bacteria in relation to the dietary treatment is indicated in Fig. 5 by the size of objects. As can be seen in Fig. 5a the relative abundance of Vibrionales is significantly higher in fish fed the 2^nd^ Feeding Diet X compared to diet Y (*P* = 0.040; see Table S1) and diet Z (*P* = 0.000), and also significantly higher when fed diet Y compared to diet Z (*P* = 0.000). In contrast, the relative abundance of Lactobacillales (Fig. 5b) was significantly higher in fish fed the plant-based 2^nd^ Feeding Diet Z compared to diet X (*P* = 0.000), as well as when compared diet Y to diet X (*P* = 0.000).

## Discussion

### Diet-type shapes the gut microbiome of juvenile brown trout

The results of this experiment demonstrate that the inclusion of plant-based ingredients into first feeding diets for brown trout reproduced from wild stocks has a significant effect on the gut microbial composition. The inclusion levels of plant-proteins or fishmeal significantly enhanced specific phyla. This has already been demonstrated in previous studies with rainbow trout^12,15,16^ and is corroborated here for brown trout. In the current experiment, Proteobacteria and Fusobacteria were significantly promoted by fishmeal present in the diet. Plant-based diets in contrast, significantly enhanced the relative abundance of Firmicutes and Bacteroidetes. The majority of bacteria found in the intestinal samples of brown trout are similar to phyla found in other salmonid species^43,44^. Proteobacteria, Firmicutes, Bacteroidetes, Fusobacteria and Actinobacteria were the most abundant phyla in samples of all treatments. The most common bacteria found in this study is Photobacterium, which is a known intestinal bacterium of carnivorous fish^45^. It is known that Bacteroidetes ferment oligosaccharides from plant material^46^, although this is mainly the case for carp. Firmicutes also incorporate several groups of lactic acid bacteria (LAB) and fermentative processes. The relative abundance of Lactobacillales, for example, significantly separated the microbiomes of brown trout that were fed a diet including plant-based proteins from those ones fed exclusively fishmeal, which has also been observed in Atlantic salmon^13^. Bifidobacteriales, in addition, were found to be significantly enhanced in brown trout fed the plant-based diets Y and Z. It is known from humans that *Bifidobacteria*, belonging to the order Bifidobacteriales, can have several physiological effects, such as an additional source of vitamins which positively affects the immune system or – in contrast – the production of lipopolysaccharides which can induce inflammation^47^. However, in an *in vitro-*study with Atlantic salmon^48^ it was demonstrated that lactic acid bacteria, isolated from the gut, can inhibit the growth of three important fish pathogens. Additionally, during the first feeding period the relative abundance of Verrucomicrobia was significantly increased in trout fry fed diet Z. Bacteria of this phylum are associated with fermentation processes^46^ and have also been observed in Atlantic salmon fed soybean protein concentrate^49^. The present experimental setup is not sufficient to investigate the metabolic contribution of the fish microbiome; nevertheless, specific bacterial groups significantly associated with one of the three experimental diets can exhibit characteristic properties, which could interact with the digestive capacity of brown trout. From several investigations in fish^10^ and in humans^47^ it is known that metabolites excreted by the bacterial gut community extensively contribute to the host metabolism. These metabolites have several functions, for example inhibitory effects against colonising pathogens^50^, but also the secretion of digestive enzymes^10^.

### The contribution of specific bacterial groups to the microbial community

NMDS analysis further demonstrates a separation of the intestinal microbiome by the three experimental diets. The bacterial communities of fish fed the mixed diet Y are always located between those of fish fed the fishmeal diet X and the plant-based diet Z. These results align well with previous results obtained with rainbow trout^16^ and with those of a comparative study on bacterial communities from different freshwater species^51^: intestinal microbiomes of those species were significantly separated by trophic status (i.e. carnivorous, herbivorous, omnivorous, filter feeders). Furthermore, Principal Coordinate Analysis (PCoA) based on unweighted UniFrac distance matrix indicated a similar position of microbiomes from omnivorous fish between those from carnivorous and herbivorous individuals in the ordination space. Thus, it might be possible that the brown trout of the present study undergo a ‘temporary’ trophic shift evoked by the diet-type. It has additionally been hypothesised that the 1^st^ Feeding Diet would have a permanent effect on the subsequent bacterial community formation in guts of early brown trout fry. However, in the PCA-based analysis no permanent effects of the 1^st^ Feeding Diet on the intestinal microbiome were observed. Instead, fishmeal and plant-protein based diets again formed specific corresponding bacterial communities during every feeding period. In contrast to the continously fed diets X and Y, diet Z provoked a different gut microbial composition in brown trout between day 61 and day 109. As discussed in Michl *et al*.^20^ the growth performance of trout continuously fed diet Z was significantly reduced compared to fish continuously fed diet X and Y, although growth performance was equal during the first feeding period. The impact on the intestinal microbiome might be the result of cumulative anti-nutritional effects formed over time and possibly related to the developmental status of the juvenile fish. Anti-nutritive effects also impact voluntary feed intake^52,53^, which in turn provokes reduced intestinal passage time, and starvation periods can significantly affect the intestinal microbiome of fish^54^. In contrast, as demonstrated by the PCA-based analysis, the microbiomes of trout at the end of the second feeding period sigificantly cluster by the 2^nd^ Feeding Diet independent of the 1^st^ Feeding Diet. Thus, the discrepancy between the two sampling days might result from the early microbiome observed on day 61, which can also be seen in Fig. 3. However, a comprehensive explanation of this finding based on our data remains speculative. Interestingly, the overall dietary effect on the intestinal microbiome is very strong, even though it is known that the host genetic background can substantially influence the bacterial composition^18^, and the experimental animals are offspring of wild fish. The results of the PCA compare well with overall findings. Moreover, the analysis strongly confirms the importance of individual bacterial groups on the formation of bacterial community structures. The orders Vibrionales, Lactobacillales, Clostridiales and Bifidobacteriales significantly separated the dietary groups according to the level of fishmeal or plant-based proteins included in the diets. As indicated already by the relative abundance of bacterial phyla, orders incorporating lactic acid producing bacteria, such as Lactobacillales and Bifidobacteriales, are mainly found in fish fed plant-based proteins, which matches earlier findings^55^. Vibrionales and Clostridiales on the other hand are relatively more abundant in fish fed fishmeal-containing diets; Vibrionales are Gammaproteobacteria, and it was found that Proteobacteria are the dominant phylum in all functional parts of the brown trout intestine when fed a commercial diet^56^. Enterobacteriaceae, Gammaproteobacteria as well, have been identified as the predominant family in the intestine of wild juvenile sea trout (*Salmo trutta trutta*)^57^.

### Diversity of the intestinal microbiome

The diversity indices calculated for individual fish were investigated for dietary effects. From the results presented in Fig. 2 it is obvious that within a treatment the individual variance of diversity indices is very high and thus statistically significant differences difficult to measure. This high inter-individual variance could reflect the various genetic backgrounds and the unknown gender of brown trout fry reproduced from wild fish. It has been demonstrated for chicken that host genotype as well as host gender significantly influence the bacterial gut community^58^. Significant associations between the microbial community and the genetic variation of individuals have also been found in humans^59^. Furthermore, it was very difficult to amplify the 16S rRNA gene in brown trout samples of the present study and thus, the number of observed OTUs was not yet exhausted in several samples as could be concluded from the rarefaction curves. Holben *et al*.^60^ compared the microbiomes of pen-raised salmon from Scotland and from Norway with wildly caught Scottish salmon, and *Mycoplasma* accounting for about 96% of all bacteria was identified from wild salmon and *Acinetobacter* for about 55% of the bacteria found in salmon of the Norwegian facility. In addition, Dehler *et al*.^61^ observed poor PCR amplification results in several intestinal samples of Atlantic salmon parr, possibly due to PCR-inhibitors with three phyla accounting for more than 80% of all sequences in the remaining samples. As can be concluded from the Chao1 richness estimator in the present study (Fig. 2b), species richness changed not significantly with time and with the amount of fishmeal used in the second feeding diets. Additionally, the analysis of pooled data after the second feeding period of the current experiment revealed that Shannon diversity was significantly increased in gut samples of fish fed the plant-based diet compared to gut samples from individuals fed the fishmeal diet. Ley *et al*.^62^ studied the co-evolution of mammals and their indigenous microbial communities and found an increasing bacterial diversity from carnivory to herbivory, which indicated a co-diversification of the intestinal microbiome with its host.

## Conclusion

To the authors’ knowledge, this is the first study providing insight into the effects of plant-based diets on the intestinal microbiome of juvenile brown trout reproduced from wild fish. The results confirm the strong influence of the feeding regime on the bacterial community structure in intestines of salmonids and demonstrate that the brown trout microbiome very well aligns with the bacterial communities found in other salmonid species. Similar to rainbow trout, for example, Proteobacteria, Firmicutes, Bacteroidetes, Fusobacteria and Actinobacteria were the dominant phyla of the brown trout gut microbiome. However, the feeding regime at first feeding induced no permanent shape of the bacterial community. After an applied diet-change, the microbiome formation proceeded again according to the fed diet-type, indicating a high plasticity of the microbiome towards dietary changes. Alpha diversity was not significantly affected by the inclusion of plant-derived proteins, but the general evenness was relatively low, indicating a high contribution of a few individual bacterial groups to the microbial community. It was also observed that certain bacterial groups associated with a specific teleost feeding strategy were significantly enhanced, when the typical diet was fed: Vibrionales and Clostridiales were associated with fishmeal diets, Lactobacillales and Bifidobacteriales with plant-based diets.

## Supplementary information

Supplementary

## Data Availability

All raw sequences used in this study are stored at the Sequence Read Archive (S.R.A.) and can be accessed via the S.R.A. accession number SRP111048 or the BioProject ID PRJNA392980 (https://www.ncbi.nlm.nih.gov/Traces/study/?acc=SRP111048).
