# Supplementary material for: A diet-change modulates the previously established bacterial gut community in juvenile brown trout (Salmo trutta)
Source: Sci Rep. 2019 Feb 20;9:2339. doi: 10.1038/s41598-019-38800-7 (PMC6382790; doi:10.1038/s41598-019-38800-7)
Supplement: Supplementary file 1 — Supplementary [file 41598_2019_38800_MOESM1_ESM.pdf]

Supplementary

A diet-change modulates the previously established bacterial gut community in juvenile brown trout (*Salmo trutta*)

Stéphanie C. Michl[1,2,3]*, Matt Beyer[4], Jenni-Marie Ratten[4], Mario Hasler[5], Julie LaRoche[4], Carsten Schulz[1,2]

[1] Gesellschaft für Marine Aquakultur mbH (GMA) Büsum, Büsum, Germany

[2] Department of Marine Aquaculture, Institute of Animal Breeding and Husbandry, Christian-Albrechts-Universität zu Kiel, Kiel, Germany

[3] GEOMAR Helmholtz Centre for Ocean Research Kiel, Kiel, Germany

[4] Department of Biology, Dalhousie University, Halifax, Canada

[5] Lehrfach Variationsstatistik, Christian-Albrechts-Universität zu Kiel, Kiel, Germany

Supplementary Figure S1

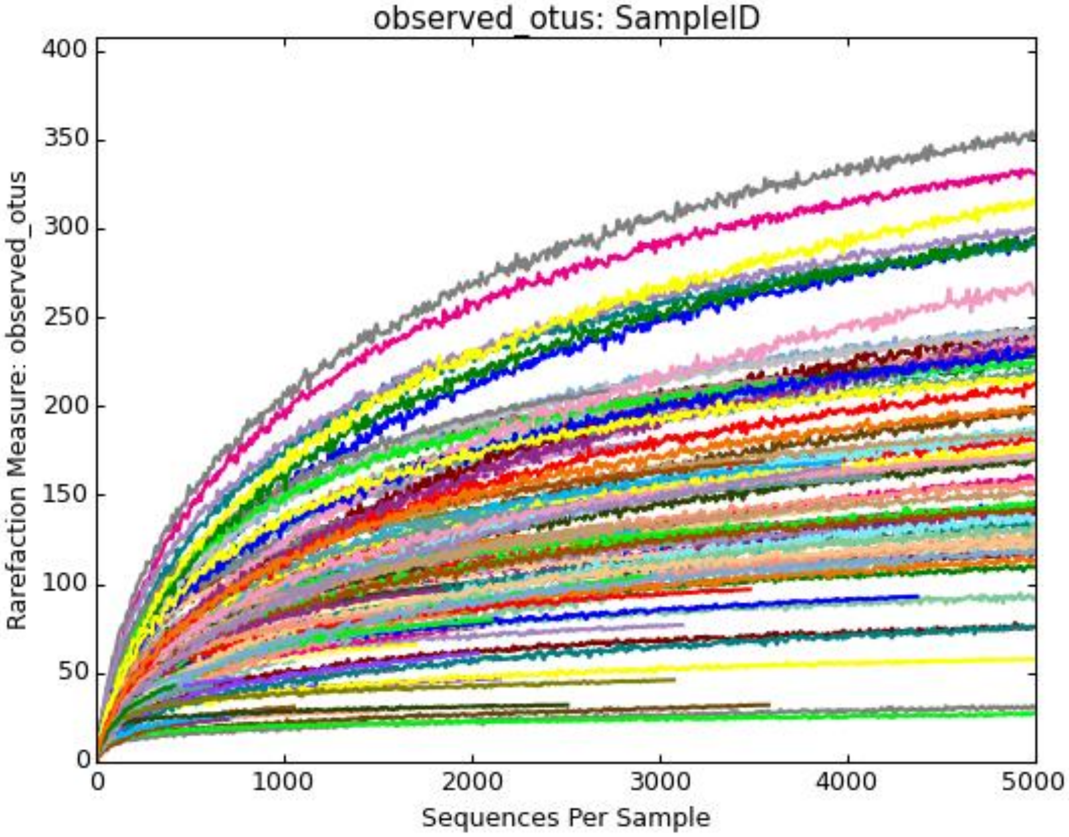

Figure S1. Rarefaction curves of observed OTUs from individual brown trout gut samples. Rarefaction curves were generated using the QIIME script alpha_rarefaction.py.

Supplementary Table S1

Table S1. Presented here are the P-values and effect sizes of the statistical analysis described in materials & methods.

1st Feeding Period

Relative abundance of top 5 phyla

	comparison	effect size	*P*-value (adj.)
Proteobacteria	Y - X	-0.315	0.006
	Z - X	-0.277	0.108
	Z - Y	0.037	0.915
Firmicutes	Y - X	0.333	0.007
	Z - X	-0.069	0.548
	Z - Y	-0.402	0.003
Bacteroidetes	Y - X	0.001	0.998
	Z - X	0.110	0.183
	Z - Y	0.109	0.175
Fusobacteria	Y - X	-0.006	0.908
	Z - X	-0.048	0.046
	Z - Y	-0.042	0.036
Actinobacteria	Y - X	0.003	0.417
	Z - X	0.160	0.427
	Z - Y	0.157	0.438

PCA / Mixed models [1]

	comparison	effect size	*P*-value (adj.)
PC1	Y - X	-0.161	0.899
	Z - X	0.793	0.139
	Z - Y	0.954	0.003
PC2	Y - X	0.037	0.899
	Z - X	0.215	0.831
	Z - Y	0.178	0.887

Loadings of top 10 bacterial orders on PC1

order	loading	order	loading
Vibrionales	-0.6412	Rhodobacterales	0.3690
Lactobacillales	-0.2700	Rhodospirillales	0.2080
Clostridiales	-0.2327	Rhizobiales	0.2059
Fusobacteriales	-0.1941	Verrucomicrobiales	0.2055
Alteromonadales	-0.1735	Saprospirales	0.1574

[1] Please consider that the mixed models following PCA were calculated based on transformed, rotated pseudo variables from the PCA.

2nd Feeding Period

Relative abundance of top 5 phyla

	comparison	effect size	*P*-value (adj.)		comparison	effect size	*P*-value (adj.)		comparison	effect size	*P*-value (adj.)
**Proteobacteria**	XY - XX	-0.119	0.702	**Bacteroidetes**	XY - XX	0.024	1.000	**Actinobacteria**	XY - XX	-0.029	0.937
	XZ - XX	-0.176	0.477		XZ - XX	0.078	0.872		XZ - XX	-0.027	0.949
	XZ - XY	-0.057	0.970		XZ - XY	0.055	0.987		XZ - XY	0.001	1.000
	YY - YX	-0.217	0.162		YY - YX	-0.018	0.998		YY - YX	0.009	0.890
	YZ - YX	-0.382	0.005		YZ - YX	0.071	0.862		YZ - YX	0.003	0.987
	YZ - YY	-0.165	0.285		YZ - YY	0.090	0.691		YZ - YY	-0.006	0.976
	ZY - ZX	-0.283	0.000		ZY - ZX	0.114	0.191		ZY - ZX	0.006	0.910
	ZZ - ZX	-0.446	0.000		ZZ - ZX	0.145	0.085		ZZ - ZX	0.001	1.000
	ZZ - ZY	-0.163	0.006		ZZ - ZY	0.031	0.999		ZZ - ZY	-0.004	0.881
Pooled 1st Feeding	Y - X	-0.206	0.000	Pooled 1st Feeding	Y - X	0.040	0.485	Pooled 1st Feeding	Y - X	-0.005	0.900
	Z - X	-0.335	0.000		Z - X	0.098	0.024		Z - X	-0.008	0.745
	Z - Y	-0.128	0.004		Z - Y	0.058	0.341		Z - Y	-0.003	0.650
**Firmicutes**	XY - XX	0.136	0.634	**Fusobacteria**	XY - XX	-0.008	0.938				
	XZ - XX	0.138	0.813		XZ - XX	-0.024	0.093				
	XZ - XY	0.002	1.000		XZ - XY	-0.016	0.051				
	YY - YX	0.251	0.030		YY - YX	-0.021	0.333				
	YZ - YX	0.328	0.037		YZ - YX	-0.037	0.016				
	YZ - YY	0.077	0.982		YZ - YY	-0.017	0.050				
	ZY - ZX	0.176	0.091		ZY - ZX	-0.026	0.030				
	ZZ - ZX	0.318	0.005		ZZ - ZX	-0.034	0.003				
	ZZ - ZY	0.142	0.601		ZZ - ZY	-0.009	0.560				
Pooled 1st Feeding	Y - X	0.188	0.000	Pooled 1st Feeding	Y - X	-0.018	0.003				
	Z - X	0.261	0.000		Z - X	-0.032	0.000				
	Z - Y	0.074	0.402		Z - Y	-0.014	0.000				

PCA / Mixed models

	diet comparison	effect size	*P*-value (adj.)
PC1	XY - XX	-0.023	1.000
	XZ - XX	0.315	0.753
	XZ - XY	0.338	0.489
	YY - YX	0.107	0.999
	YZ - YX	0.466	0.030
	YZ - YY	0.359	0.151
	ZY - ZX	0.410	0.057
	ZZ - ZX	0.456	0.004
	ZZ - ZY	0.046	1.000
Pooled 1st Feeding	Y - X	0.165	0.477
	Z - X	0.412	0.000
	Z - Y	0.248	0.020
PC2	XY - XX	0.214	0.800
	XZ - XX	0.258	0.714
	XZ - XY	0.044	1.000
	YY - YX	0.344	0.055
	YZ - YX	0.454	0.018
	YZ - YY	0.110	1.000
	ZY - ZX	0.284	0.009
	ZZ - ZX	0.449	0.000
	ZZ - ZY	0.165	0.781
Pooled 1st Feeding	Y - X	0.281	0.000
	Z - X	0.387	0.000
	Z - Y	0.106	0.707

Loadings of top 10 bacterial orders on PC1

order	loading	order	loading
Vibrionales	-0.6530	Rhodobacterales	0.3363
Clostridiales	-0.2134	Cytophagales	0.3287
Fusobacteriales	-0.1783	Saprospirales	0.2692
Lactobacillales	-0.1144	Burkholderiales	0.2562
Alteromonadales	-0.0701	Flavobacteriales	0.2354

Loadings of top 10 bacterial orders on PC2

order	loading	order	loading
Vibrionales	-0.3625	Lactobacillales	0.8514
Alteromonadales	-0.1618	Bacillales	0.0729
Rhodobacterales	-0.1525	Bifidobacteriales	0.0673
Cytophagales	-0.1282	Actinomycetales	0.0664
Burkholderiales	-0.1080	Enterobacteriales	0.0638

Significant differences of bacterial orders contributing to PC1

Pooled 1st Feeding	diet comparison	effect size	*P*-value (adj.)
Vibrionales	Y - X	-0.178	0.040
	Z - X	-0.391	0.000
	Z - Y	-0.213	0.000
Clostridiales	Y - X	-0.059	0.278
	Z - X	-0.145	0.000
	Z - Y	-0.086	0.001
Fusobacteriales	Y - X	-0.046	0.616
	Z - X	-0.113	0.000
	Z - Y	-0.067	0.000
Lactobacillales	Y - X	0.252	0.000
	Z - X	0.300	0.000
	Z - Y	0.048	5.734
Alteromonadales	Y - X	-0.057	0.052
	Z - X	-0.082	0.003
	Z - Y	-0.024	4.166

Significant differences of bacterial orders contributing to PC2

Pooled 1st Feeding	diet comparison	effect size	*P*-value (adj.)
Vibrionales	Y - X	-0.178	0.040
	Z - X	-0.391	0.000
	Z - Y	-0.213	0.000
Alteromonadales	Y - X	-0.057	0.052
	Z - X	-0.082	0.003
	Z - Y	-0.024	4.166
Rhodobacterales	Y - X	0.056	2.169
	Z - X	0.085	0.610
	Z - Y	0.030	6.589
Lactobacillales	Y - X	0.252	0.000
	Z - X	0.300	0.000
	Z - Y	0.048	5.734
Bifidobacteriales	Y - X	0.035	0.004
	Z - X	0.022	0.113
	Z - Y	-0.013	3.553
